# Case Report: Delayed diagnosis of pancreatic solid pseudopapillary neoplasm in a boy initially misdiagnosed as traumatic retroperitoneal hematoma

**DOI:** 10.3389/fped.2026.1899711

**Published:** 2026-09-16

**Authors:** Zhaowei Zhou, Rifang Pan, Junzhong Li, Zhaopan Li, Zhihua Xiao

**Affiliations:** Department of Pediatric Surgery, Yulin First People's Hospital, Yulin, Guangxi, China

**Keywords:** delayed diagnosis, hematoma, solid pseudopapillary neoplasm, spleen-preserving distal pancreatectomy, trauma

## Abstract

Solid pseudopapillary neoplasm (SPN) of the pancreas is a rare pancreatic tumor with low-grade malignant potential. It predominantly occurs in adolescent girls and young women but can also affect boys. SPN may contain hemorrhagic or cystic components that can be mistaken for a post-traumatic hematoma when patients present after abdominal injury. We report a 12-year-old boy whose pancreatic tail lesion was initially interpreted as a traumatic retroperitoneal hematoma after minor blunt trauma at age 7. Ultrasound-guided drainage yielded hemorrhagic fluid with non-elevated amylase levels and negative bacterial culture; however, cytological examination was not documented. The lesion persisted, and scheduled follow-up was subsequently discontinued. Approximately 4.5 years later, repeat evaluation demonstrated interval enlargement from approximately 5.6 cm to 10 cm. Imaging showed a complete thin capsule, mixed solid-cystic architecture, coarse calcification, and progressive enhancement, features that were not compatible with a resolving hematoma. After multidisciplinary reassessment, SPN was considered the most likely diagnosis. Laparoscopic spleen-preserving distal pancreatectomy was successfully performed, with an estimated blood loss of 30 mL. Histopathological examination confirmed SPN, with a Ki-67 index of approximately 2%, a negative stapled margin, and no tumor involvement in the sampled adjacent pancreatic or fibrofatty tissue. The patient did not develop a clinically relevant postoperative pancreatic fistula. During approximately 11 months of postoperative surveillance, no recurrence was detected. This case emphasizes that a presumed post-traumatic hematoma should be reassessed when it persists or enlarges. It also supports the feasibility of minimally invasive spleen-preserving resection in carefully selected pediatric patients.

## Introduction

Solid pseudopapillary neoplasm (SPN) is an uncommon epithelial tumor of the pancreas characterized by low-grade malignant potential and favorable long-term outcomes following complete surgical resection (1, 2). Pediatric SPN is rare. One multicenter study identified 23 pediatric cases, and subsequent registry-based and single-institution series have also reported limited sample sizes (3–7). Across larger reviews, SPN has consistently been described as an indolent neoplasm with a marked female predominance and excellent postoperative survival (1, 2, 5–7). For lesions arising in the pancreatic body or tail, distal pancreatectomy remains the standard surgical strategy. Preservation of the spleen is preferred when it can be achieved without compromising oncological safety or technical feasibility (1–3, 8–10).

Despite these generally favorable features, preoperative diagnosis of SPN can be difficult. Symptoms are frequently absent or nonspecific, and radiological findings may overlap with those of other cystic, hemorrhagic, or post-traumatic pancreatic lesions (2, 4, 11). Intratumoral hemorrhage is a recognized feature of SPN and may closely resemble traumatic hematoma, especially in children who present after abdominal injury (11). In such cases, the history of trauma may strongly influence the initial diagnostic interpretation and obscure an underlying neoplasm, thereby contributing to delayed diagnosis (12).

We report a pediatric case of SPN in a 12-year-old boy whose pancreatic tail lesion persisted after drainage of hemorrhagic fluid and enlarged from approximately 55 × 45 mm to 10 cm during a diagnostic interval of approximately 4.5 years. Although the tumor was large at the time of definitive treatment, laparoscopic spleen-preserving distal pancreatectomy was completed successfully. The case is clinically notable because of the trauma-associated initial presentation, prolonged persistence of a presumed hematoma, substantial interval growth, and successful minimally invasive organ-preserving resection.

## Case presentation

At age 7, the patient presented after minor blunt abdominal trauma. Initial ultrasonography showed a 55 × 45 mm mixed solid-cystic lesion in the pancreatic tail, without main pancreatic duct dilatation or intralesional Doppler flow. Multiphase CT further demonstrated a well-demarcated, predominantly cystic mass measuring 5.6 × 5.2 × 4.7 cm, with mild and relatively homogeneous enhancement of the wall and no enhancement within the cystic component. On the basis of the trauma history and imaging appearance, the lesion was interpreted as either a traumatic retroperitoneal hematoma or a pancreatic pseudocyst. Ultrasound-guided drainage was performed using an 8.5-F pigtail catheter and yielded dark-red hemorrhagic fluid. The fluid amylase level was not elevated, and bacterial culture was negative. However, no cytological examination, histological assessment, or cell-block analysis was documented. Follow-up ultrasonography showed that the lesion remained present, measuring 46 × 52 mm on day 3 after drainage, 48 × 45 mm on day 7, 42 × 42 mm two days after drain removal, and 47 × 34 mm 16 days after drain removal. These findings indicated that the lesion did not undergo the sustained reduction expected for a resolving hematoma. Nevertheless, regular follow-up was subsequently discontinued.

Approximately 4.5 years after the initial presentation, the patient noticed a palpable mass in the left upper abdomen. One month later, at age 12, he was readmitted with abdominal pain and vomiting after another episode of minor blunt abdominal trauma. He had no fever, jaundice, hematemesis, or rectal bleeding. Physical examination showed a soft abdomen, mild tenderness in the left upper quadrant, and no signs of peritoneal irritation.

Laboratory evaluation revealed a white blood cell count of 12.29 × 10^9^/L, neutrophils 68.4%, hemoglobin 139 g/L, platelet count 353 × 10^9^/L, serum amylase 80 U/L, serum lipase 14.8 U/L, and C-reactive protein 0.45 mg/L. Serum CA19-9, CA72-4, CA15-3, and CA125 levels were within their respective reference ranges. Neuron-specific enolase was elevated at 69.30 ng/mL. Repeat ultrasonography revealed a 102 × 73 × 82 mm mixed solid-cystic retroperitoneal mass, with punctate internal and linear peripheral blood-flow signals and a central echogenic calcification. Multiphase CT showed an approximately 10-cm encapsulated mixed solid-cystic mass arising from the pancreatic body-tail. The mass contacted and displaced the posterior wall of the stomach and the adjacent colon. It also compressed the spleen, left kidney, and left adrenal region, but there was no radiological evidence of direct invasion. The splenic artery and vein coursed along the posteromedial aspect of the tumor and were stretched and compressed; however, both vessels remained continuous and patent, without encasement or thrombosis. The main pancreatic duct was not dilated. There was no ascites, capsular disruption, tumor rupture, lymphadenopathy, or distant metastasis. On unenhanced CT, coarse calcification was evident within the tumor. After contrast administration, the solid components and capsule showed mild-to-moderate heterogeneous enhancement in the arterial phase, followed by persistent and progressively more conspicuous enhancement in the venous and delayed phases. The cystic, hemorrhagic, and necrotic components showed no enhancement. Representative CT images from the initial and current presentations are shown in Figure 1. MRI or MRCP was not performed, and the reason was not documented in the available medical record.

**Figure 1:**
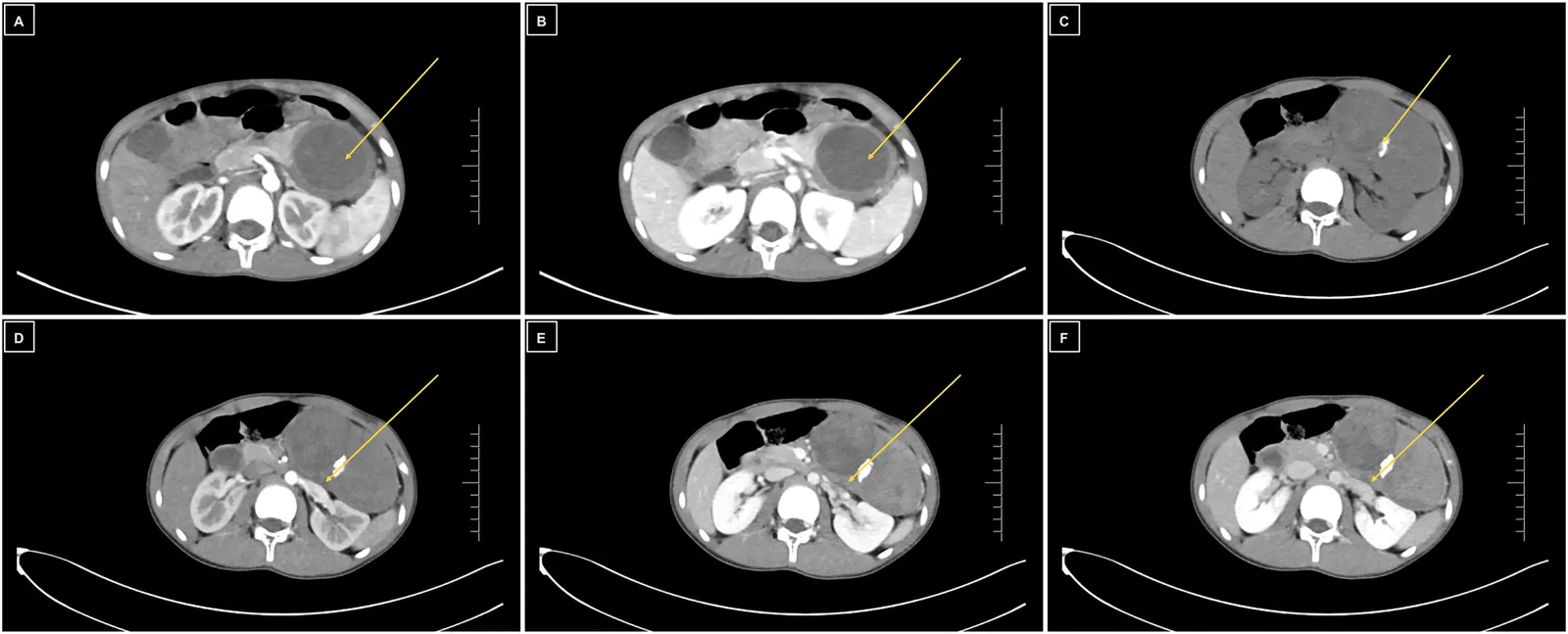
Representative CT images from the initial and current presentations. **(A,B)** Initial arterial- and venous-phase images show a 5.6-cm, well-circumscribed, predominantly cystic lesion in the pancreatic tail, with mild wall and peripheral enhancement. **(C–F)** Images obtained approximately 4.5 years later show an enlarged 10-cm encapsulated mixed solid-cystic mass on unenhanced, arterial-phase, venous-phase, and delayed-phase CT, respectively. The yellow arrows indicate the lesion in **(A,B)**, coarse intratumoral calcification in **(C)**, and heterogeneously enhancing solid tissue in **(D-F)**. The solid components and capsule show persistent progressive enhancement, whereas the cystic and necrotic regions remain non-enhancing.

Given the long-term persistence and marked enlargement of the lesion, a structured diagnostic reassessment was undertaken. The differential diagnosis included SPN, pancreatic pseudocyst, organizing hematoma, pancreatoblastoma, pancreatic neuroendocrine tumor, acinar cell carcinoma, and other cystic pancreatic or retroperitoneal lesions. The approximately 4.5-year persistence, substantial interval growth, complete capsule, mixed solid-cystic morphology, coarse calcification, and heterogeneous progressive enhancement were not compatible with a resolving hematoma. These features instead strongly favored SPN. No preoperative fine-needle aspiration biopsy was performed during the current admission.

The longitudinal clinical and imaging findings were reviewed by a multidisciplinary team comprising pediatric surgery, radiology, abdominal tumor specialists, hepatobiliary surgery, and pediatric medicine. The team favored a diagnosis of SPN. Surgical resection was recommended because the tumor was large, technically resectable, and highly suspicious for SPN. Histopathological and immunohistochemical analysis of the resected specimen subsequently confirmed the diagnosis.

On hospital day 5, the patient underwent laparoscopic spleen-preserving distal pancreatectomy, adhesiolysis, and splenic repair. Intraoperative exploration revealed a 10 × 8 × 7 cm encapsulated tumor in the pancreatic body-tail region. The tumor adhered to adjacent bowel and the lower pole of the spleen. No peritoneal, hepatic, or pelvic nodules were observed, and no enlarged peripancreatic lymph nodes were identified. The splenic artery and vein were dissected separately and preserved, and their pancreatic branches were clipped. The pancreas was divided approximately 1 cm proximal to the right margin of the tumor using a linear stapler, and the pancreatic stump was reinforced using interrupted sutures. The spleen was preserved after repair of the lower-pole surface. Representative intraoperative findings are shown in Figure 2. The operation lasted 340 min, with an estimated blood loss of 30 mL, and no transfusion was required. Two silicone drains were placed in the lesser sac and pelvis.

**Figure 2:**
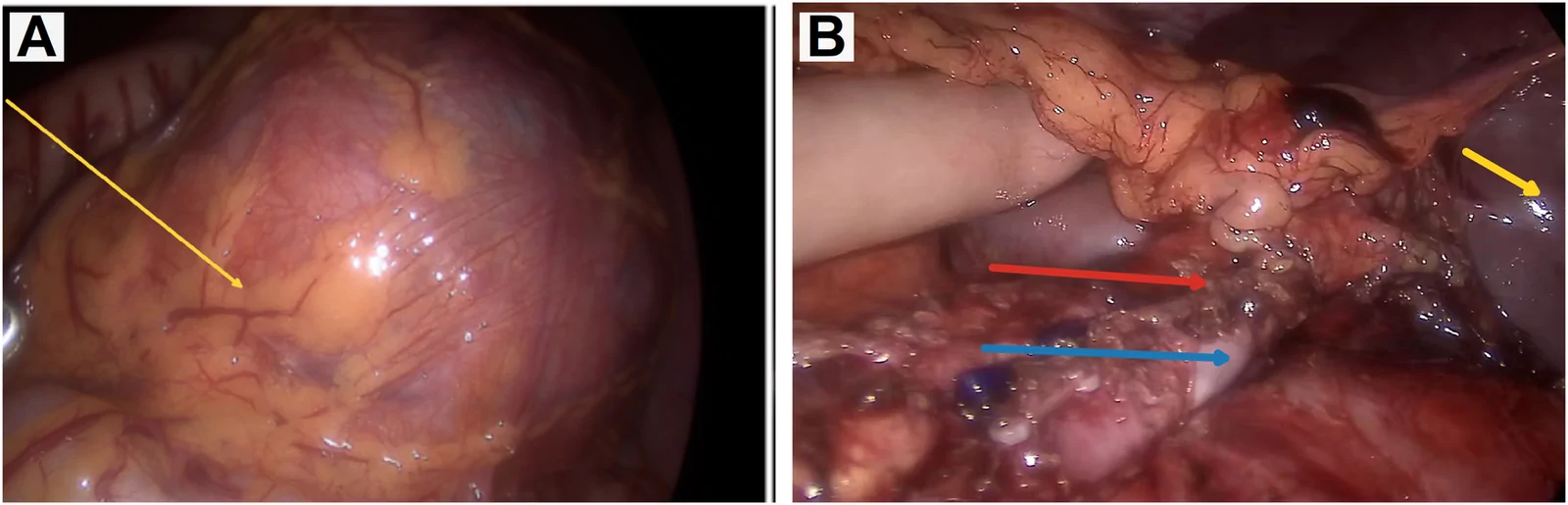
Representative intraoperative images from laparoscopic spleen-preserving distal pancreatectomy. **(A)** The yellow arrow indicates the large encapsulated tumor occupying the left upper abdomen. **(B)** After tumor resection, the yellow, red, and blue arrows indicate the preserved spleen, splenic artery, and splenic vein, respectively.

Gross examination showed a 10 × 7.4 × 5.5 cm partially encapsulated mixed solid-cystic tumor containing fibrous septa, hemorrhage, necrosis, and focal calcification/ossification. Microscopy demonstrated diffuse sheets of tumor cells and pseudopapillary structures arranged around fibrovascular cores. The tumor cells were relatively uniform, mitotic figures were rare, and no lymphovascular tumor emboli were identified. The tumor did not involve the sampled surrounding pancreatic tissue, and the submitted fibrofatty adhesion tissue was negative for tumor. The stapled pancreatic margin was free of tumor. The pancreas had been divided approximately 1 cm from the gross tumor edge, although the microscopic clearance distance was not reported. Capsular invasion and perineural invasion were not reported. No lymph nodes were submitted for pathological assessment. Immunohistochemical staining showed cytoplasmic and nuclear expression of *β*-catenin. The tumor cells were positive for vimentin, CK18, CD10, and CD56, with focal positivity for synaptophysin and weak positivity for progesterone receptor. They were negative for E-cadherin, TFE3, PAX8, pancytokeratin, CK7, chromogranin A, and SALL4. The Ki-67 proliferation index was approximately 2%. No radiological or intraoperative evidence of tumor rupture was documented. CTNNB1 molecular testing was not performed or reported; accordingly, *β*-catenin immunohistochemistry served as supportive diagnostic evidence. Overall, the findings confirmed SPN with a low proliferation index and no documented invasion of the sampled adjacent tissues.

The postoperative course was uncomplicated. Serum amylase was 80 U/L before surgery, 98 U/L on the day of surgery, 129 U/L on postoperative day 3, and 111 U/L on postoperative day 7. All values remained within the laboratory reference range. On postoperative day 2, fluid from the lesser-sac drain showed an amylase level of 8,864 U/L and a lipase level of 26,064 U/L. Because drain-fluid amylase was not measured on or after postoperative day 3, the isolated day-2 elevation could not be formally classified as a biochemical leak according to the 2016 ISGPS definition (13). The patient had no infection, no intra-abdominal collection requiring intervention, no organ failure, no reoperation, and no other clinically meaningful postoperative complication. No grade B or C postoperative pancreatic fistula occurred. The remaining lesser-sac drain was removed on postoperative day 9, and the patient was discharged the same day after a total hospital stay of 13 days. During follow-up, serum amylase remained within the reference range at approximately 1, 5, and 11 months after surgery. Serum lipase was mildly elevated at 1 month but returned to normal thereafter. Serial ultrasonography and CT showed only postoperative changes, with no evidence of tumor recurrence or persistent intra-abdominal fluid collection. The latest evaluation, performed approximately 11 months after surgery, represents short-term surveillance. Follow-up was scheduled at 2 weeks and at 1, 3, and 6 months postoperatively, followed by examinations every 6–12 months. Surveillance included tumor markers, ultrasonography, and CT when clinically indicated.

## Discussion

This case illustrates a relevant diagnostic pitfall in pediatric pancreatic disease: diagnostic anchoring after trauma. Because the patient presented after blunt abdominal injury and the initial aspirate contained dark hemorrhagic fluid, a post-traumatic hematoma was a plausible preliminary diagnosis. The main issue was not the initial interpretation, but the absence of timely reassessment when the lesion failed to follow the expected clinical course. The mass did not regress, persisted for several years, and later enlarged substantially. This course shows that a trauma-associated working diagnosis may remain unchallenged unless serial imaging findings and interval growth are critically reviewed.

SPN is a low-grade malignant pancreatic neoplasm with a favorable prognosis after complete surgical resection. Large systematic reviews have shown that SPN occurs predominantly in young women, commonly presents with abdominal pain or a palpable mass, and is associated with excellent outcomes after complete resection, with overall survival exceeding 95% in most series (1, 2, 5, 6). Although SPN mainly affects adolescent girls and young adult women, male patients can also be affected, as shown in this case (3, 5, 7). The characteristic pathological features of SPN include pseudopapillary architecture, nuclear *β*-catenin expression, and immunopositivity for CD10 and CD56 (11).

Several findings in this patient favored SPN rather than a post-traumatic hematoma. Over approximately 4.5 years, the lesion enlarged from approximately 55 × 45 mm to 10 cm and maintained a complete capsule, mixed solid-cystic morphology, and heterogeneous enhancement. This pattern was inconsistent with the expected evolution of resolving blood products. SPN presenting as a traumatic hematoma mimic, particularly in boys, has been reported previously (12). The present case is notable for the prolonged diagnostic delay, substantial enlargement after drainage of hemorrhagic fluid, persistence after a trauma-associated presentation, and successful spleen-preserving laparoscopic resection despite the final tumor size. Fine-needle aspiration or biopsy under endoscopic ultrasonographic or percutaneous guidance may provide cytological or histological evidence when the diagnosis remains uncertain (14, 15). During the initial admission, percutaneous drainage was performed because the lesion was regarded as a hematoma or pseudocyst. The hemorrhagic fluid was tested for amylase and bacterial culture, but no cytological, histological, or cell-block evaluation was documented. This limited the initial diagnostic assessment and represented a missed opportunity to reconsider the presumed traumatic lesion. During the current admission, preoperative biopsy was not performed because multiphase imaging and multidisciplinary review strongly favored a resectable SPN, for which surgical excision was required for definitive diagnosis and treatment.

In children, the differential diagnosis for a mixed solid-cystic pancreatic lesion includes pancreatic pseudocyst, organizing hematoma, pancreatoblastoma, pancreatic neuroendocrine tumor, acinar cell carcinoma, and other cystic pancreatic or retroperitoneal lesions (16, 17). In this patient, the structured reassessment favored SPN because of the lesion's long-term persistence, interval growth, encapsulation, internal solid components, and heterogeneous enhancement, all of which argued against a resolving traumatic lesion.

The surgical management was also noteworthy. The tumor was large, the pediatric abdominal cavity provided limited working space, and the previous drainage procedure likely contributed to local adhesions. Despite these technical challenges, laparoscopic spleen-preserving distal pancreatectomy was completed successfully using a Kimura-type approach. This technique preserves the splenic artery and vein together with the spleen and is particularly appropriate in pediatric patients because it avoids the long-term immunologic consequences of splenectomy (8, 9). This case supports the feasibility of this approach in carefully selected children with SPN.

This report has several limitations. The initial aspirate did not undergo documented cytological, histological, or cell-block evaluation. MRI/MRCP was not performed, and the clinical reason was not recorded. Drain-fluid amylase was measured on postoperative day 2 but not on or after postoperative day 3, precluding strict classification of a biochemical leak according to the ISGPS definition. The pathology report did not include the microscopic margin distance, explicit assessment of capsular or perineural invasion, lymph-node evaluation, or CTNNB1 molecular testing. Finally, follow-up is currently limited to approximately 11 months, which is insufficient to determine the long-term oncological outcome.

## Conclusion

SPN should be considered when a pancreatic lesion in a child persists or enlarges after a trauma-associated presentation, even when aspiration yields hemorrhagic fluid. Careful review of longitudinal imaging and multidisciplinary reassessment may help reduce prolonged diagnostic delay. In selected pediatric patients with resectable SPN, laparoscopic spleen-preserving distal pancreatectomy using the Kimura technique may provide an organ-preserving surgical option. Longer surveillance is required before the oncological outcome of this patient can be assessed.

## Data Availability

The original contributions presented in the study are included in the article. Further inquiries can be directed to the corresponding author.
